# Correction: *WormSNAP*: A software for fast, accurate, and unbiased detection of fluorescent puncta in *C. elegans*

**DOI:** 10.1371/journal.pcbi.1013953

**Published:** 2026-02-10

**Authors:** Araven Tiroumalechetty, Elisa B. Frankel, Peri T. Kurshan

In the Fluorescent puncta detection in C. elegans research subsection of the Introduction, a reference is omitted from the seventh sentence of the third paragraph.

The correct sentence is: Although some progress has been made in switching to automated identification of 2D regions of interest (ROIs) to retain spatial information [7,12] [Hulsey-Vincent et al., 2023], no widely adopted method yet exists in this subfield.

The reference is: Hulsey-Vincent H, Alvinez N, Witus S, Kowalski JR, Dahlberg C. 2023. A Fiji process for quantifying fluorescent puncta in linear cellular structures. microPublication Biology. https://doi.org/10.17912/micropub.biology.001003.

## References

[1] Tiroumalechetty A, Frankel EB, Kurshan PT. *WormSNAP*: A software for fast, accurate, and unbiased detection of fluorescent puncta in C. elegans. PLoS Comput Biol. 2025;21(10):e1013643. doi: 10.1371/journal.pcbi.1013643 41171895 PMC12599913

