# Supplementary figures and images for: WormSNAP: A software for fast, accurate, and unbiased detection of fluorescent puncta in C. elegans
Source: PLoS Comput Biol. 2025 Oct 31;21(10):e1013643. doi: 10.1371/journal.pcbi.1013643 (PMC12599913; doi:10.1371/journal.pcbi.1013643)

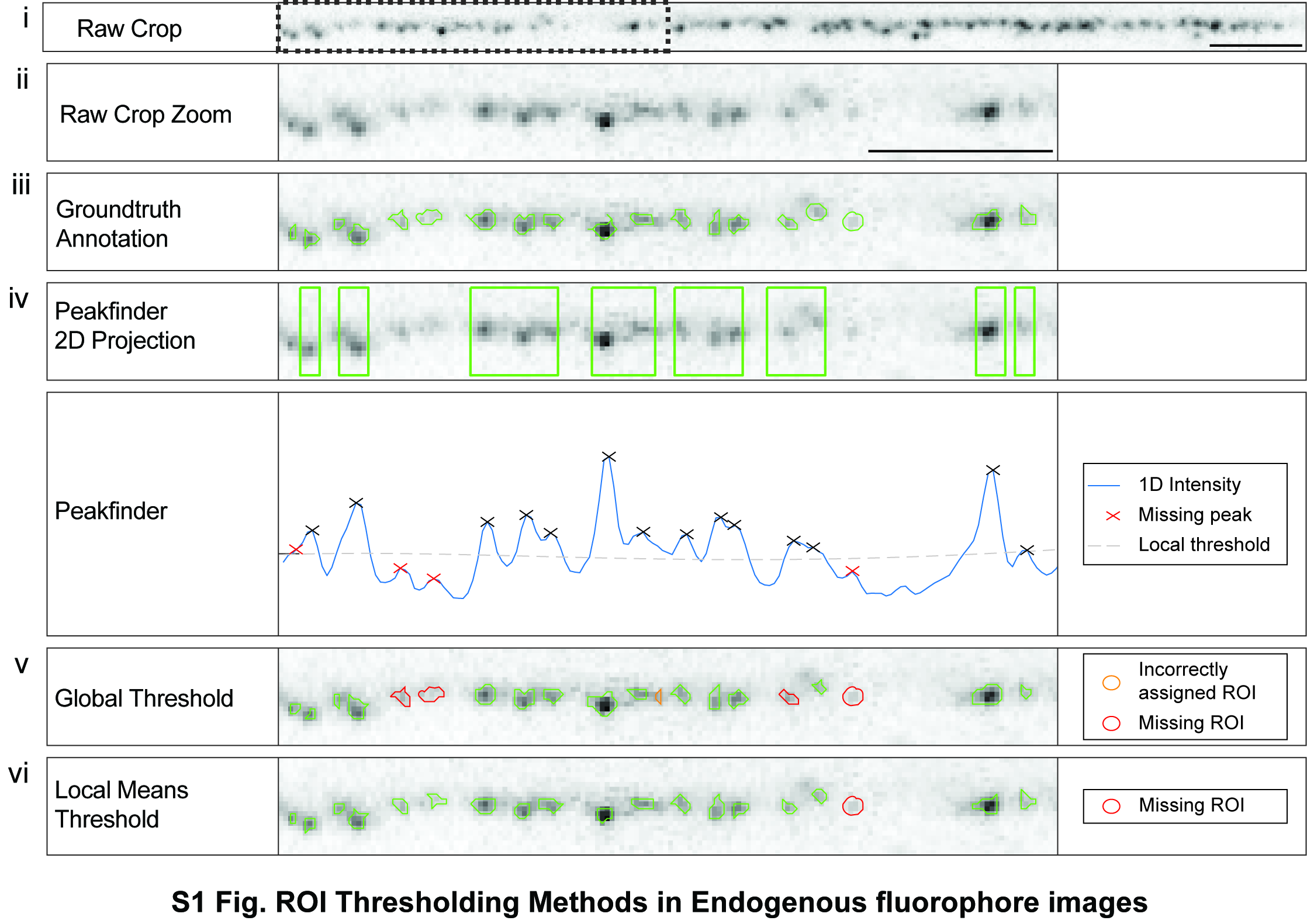

Supplement: S1 Fig — Example Crop from 100X confocal images of endogenous synaptic marker in the dorsal nerve cord (Neurexin::Skylan-S) with annotated ROIs (green) from different methods showing: i. The raw crop with a region highlighted; ii. A zoom in of the raw crop in the highlighted region; iii. 2D ROIs from the Ground Truth annotation; iv. Projected 2D ROIs from 1D peak finding algorithm, with valid peaks labeled by black Xs and unlabeled puncta labeled with red Xs; v. 2D ROIs from Global Thresholding algorithm, with erroneous puncta in orange and missing puncta highlighted in red; and vi. 2D ROIs from Local Means Thresholding algorithm, also showing missing puncta highlighted in red. The local means thresholding method demonstrates the highest fidelity to the ground truth annotation. Scale Bars shown on i and ii, 5µm. (TIF) [file pcbi.1013643.s001.tif]

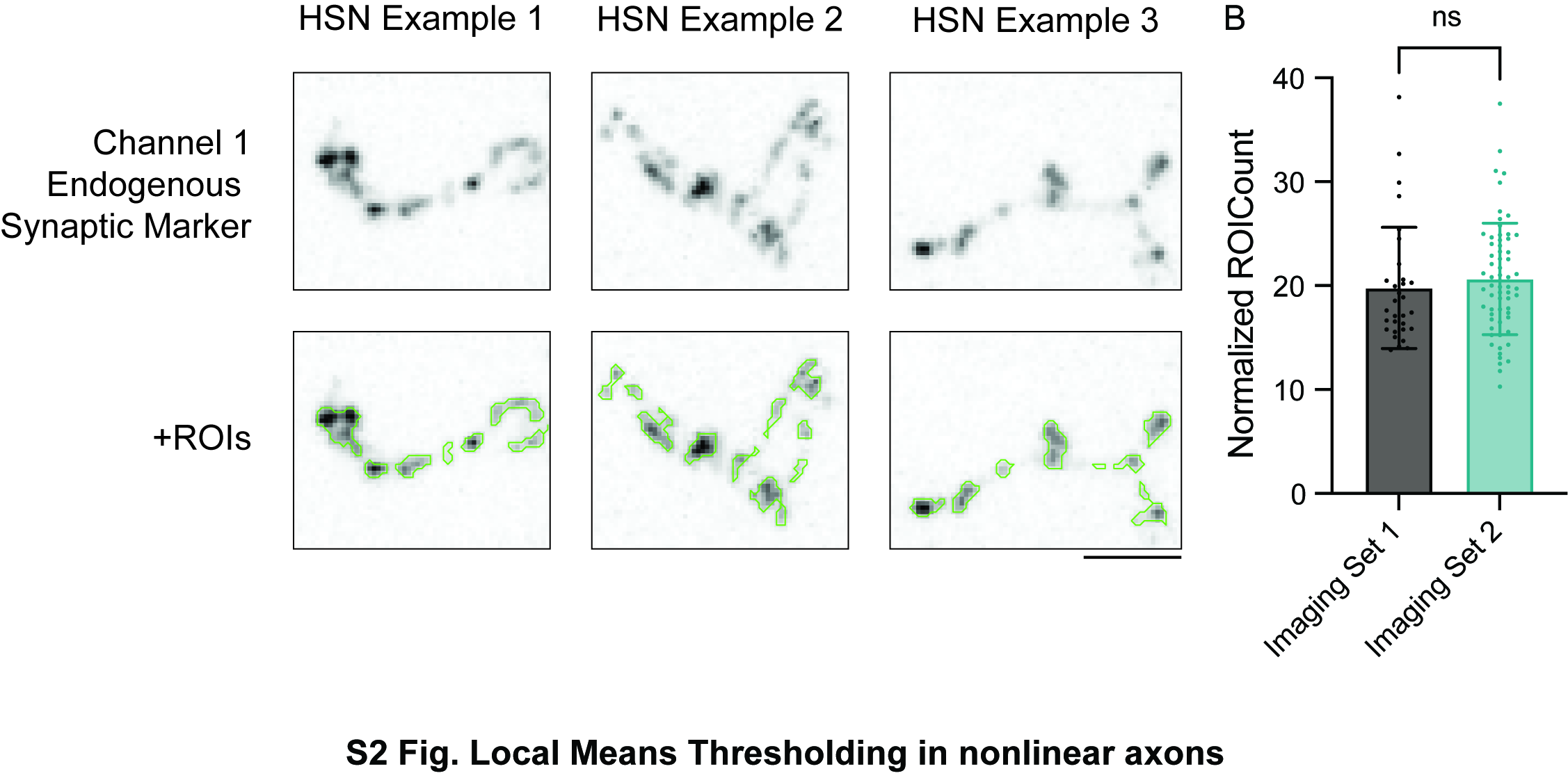

Supplement: S2 Fig — (A) Example crops of HSN synapses from worms with endogenous expression of a synaptic fluorophore (SYD-1::mScarlet). Top: Crops. Bottom: Crops with ROI overlay (green). Scale, 3 µm. (B) Quantification of number of SYD-1::mScarlet ROIs (normalized to 100µm2) in HSN synapses from two different imaging slides. The lack of significant difference between the two samples demonstrates replicability of local means thresholding in nonlinear axons. (TIF) [file pcbi.1013643.s002.tif]

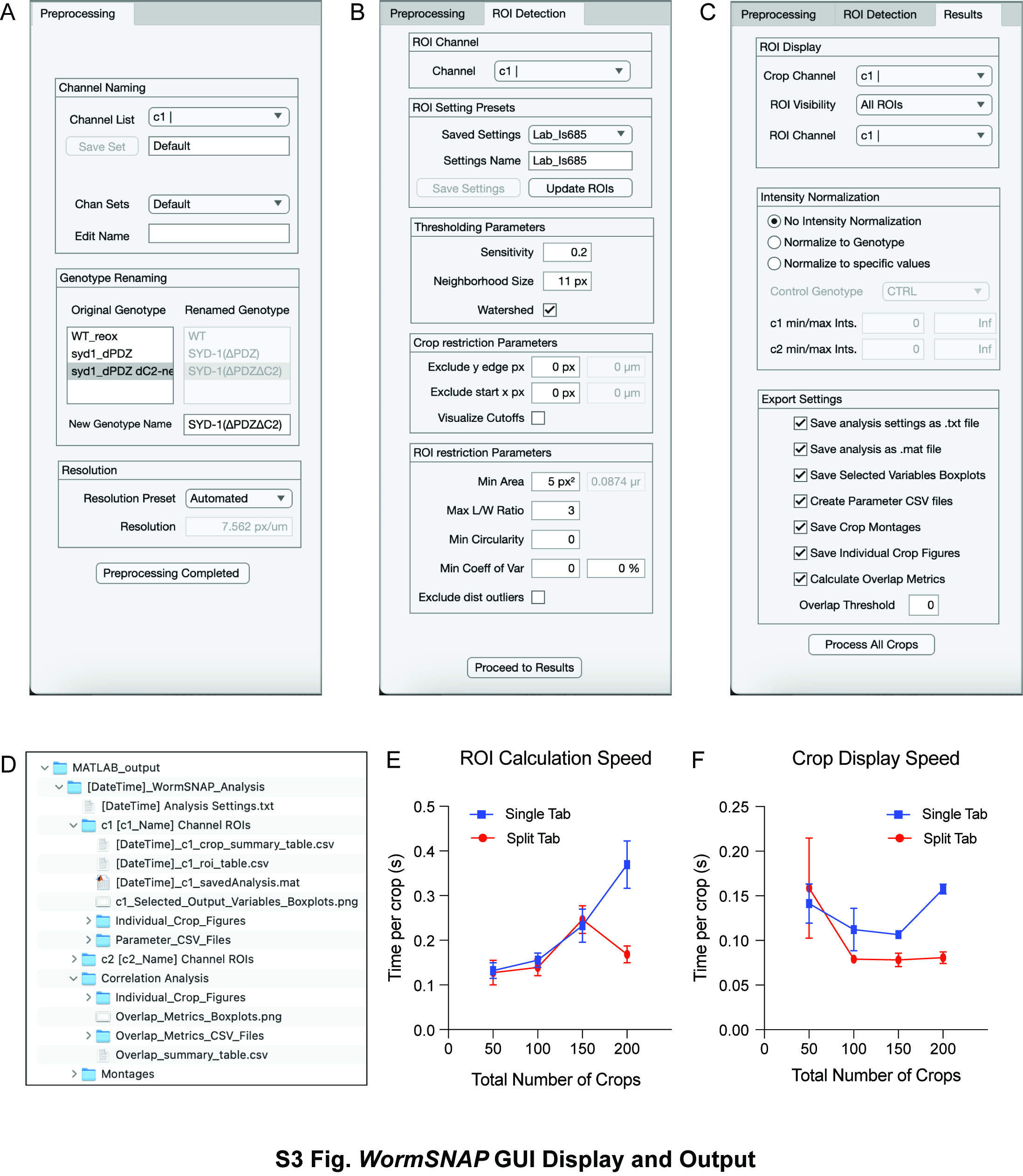

Supplement: S3 Fig — (A) Screenshot of the Preprocessing Tab in the Graphic User Interface (GUI). (B) Screenshot of the ROI Detection Tab in the GUI. (C) Screenshot of the Results Tab in the GUI. (D) Example Output folder for a 2-channel dataset generated using the output settings shown in (C). (E) Crop Display Speed, measured in seconds per crop, for datasets of various sizes displayed in a single-tab versus a split-tab. The split-tab approach limits each tab to a maximum of 50. (F) ROI Calculation speed (seconds per crop) for datasets of various sizes displayed in a single-tab versus a split-tab. (TIF) [file pcbi.1013643.s003.tif]

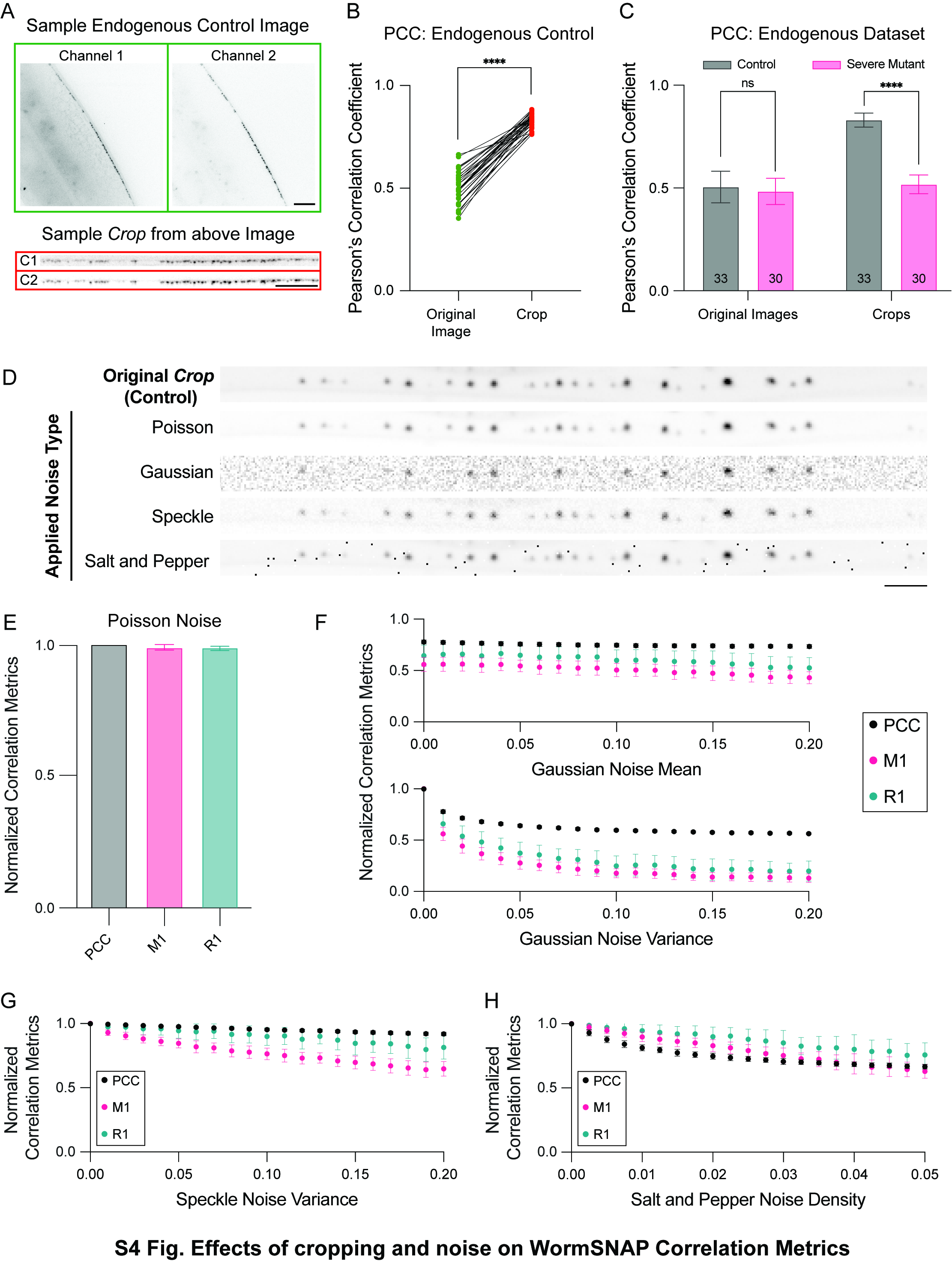

Supplement: S4 Fig — (A) Example 2 channel confocal image from a control worm from the endogenous fluorophore dataset with nrx-1::Skylan-S in Channel 1 and syd-1::mScarlet in channel 2 with the corresponding 2-channel crop. Scale Bars, 5µm. (B) Pearson’s Correlation Coefficient (PCC) for original images and resulting crops for the control worms in the endogenous fluorophore dataset (N = 33) (* = p < 0.05, ** = p < 0.01, *** = p < 0.001, **** = p < 0.0001). (C) PCC for original images and resulting crops of the control and severe clustering mutant (syd-1(∆PDZ)) from the Endogenous dataset, demonstrating that cropping images improves the PCC metric for control but not for the severe clustering mutant phenotype. Consequently, crops show a significant difference in PCC between the two strains, whereas original images do not (* = p < 0.05, ** = p < 0.01, *** = p < 0.001, **** = p < 0.0001). (D) Image of a crop from the Overexpressed Synaptic Fluorophore Dataset showing Channel 1 (CLA-1::GFP) and the same crop after different types of noise was applied to the signal. Poisson noise was generated using a Poisson function with mean equivalent to signal pixel intensity; Gaussian noise was generated using a gaussian function with mean 0 and variance 0.01; speckle noise was generated using a uniformly random noise function with mean 0 and variance 0.05; salt and pepper noise was generated using a noise density of 0.05 (5% of pixels replaced). illustrating how different noise types affect signal in crops. Scale Bar, 5µm. (E) Quantification of three Correlation Metrics – PCC (normalized to [0,1] from its original range of [-1,1), Manders’ Coefficient (M1), and ROI Overlap ratio (R1) for synthetic 2-channel images consisting of the original channel 1 of the Overexpressed Synaptic Fluorophore Dataset (N = 52) and channel 2 generated by applying Poisson noise (used to simulate shot noise) to the first channel. The mean and standard deviation of each metric was plotted. (F) Quantification of the three Co [file pcbi.1013643.s004.tif]
