# Supplementary material for: WormSNAP: A software for fast, accurate, and unbiased detection of fluorescent puncta in C. elegans
Source: PLoS Comput Biol. 2025 Oct 31;21(10):e1013643. doi: 10.1371/journal.pcbi.1013643 (PMC12599913; doi:10.1371/journal.pcbi.1013643)
Supplement: S1 Table — (DOCX) [file pcbi.1013643.s005.docx]

| **Strain** | **Genotype** | **Figures** | **Notes** |
| --- | --- | --- | --- |
| TV18675 | *wyIs685 V* | 1B, 1D-G  2A-C (Control),  3, 4A, 4C, S4 C-G | *wyIs685 V*: integrated array of mig-13p::3xnovoGFP::cla-1S (referred to as CLA-1::GFP in the text) + mig-13p::TdTomato::rab-3 |
| PTK323 | *syd-1(ju82) II; wyIs685V* | 2A-C (Severe Mutant), 4B | *syd-1(ju82) II*: nonsense mutation; |
| TV22469 | *wyIs685,*  *nrx-1(wy1155) V* | 2A-C (Mild Mutant) | *nrx-1(wy1155) V*: full deletion of neurexin (nrx-1) locus using *CRIPSR* |
| PTK196 | *syd-1(kur33) II;*  *nrx-1(ox719) V* | 1C, 1E-G, 2D-F (Control), S1,  S4A-C (Control) | *syd-1(kur33) II*: endogenous syd-1::mScarlet,  *nrx-1(ox719) V*: endogenous nrx-1::skylan-S |
| PTK270 | *syd-1(kur53) II;*  *nrx-1(ox719) V* | 2D-F (Severe Mutant),  S4C (Severe Mutant) | *syd-1(kur53) II*: syd-1(∆PDZ)::mScarlet, deletion of SYD-1’s PDZ domain through *CRISPR* |
| PTK362 | *syd-1(kur75) II;*  *nrx-1(ox719) V* | 2D-F (Mild Mutant) | *syd-1(kur75) II*: syd-1(∆C2)::mScarlet,  deletion of SYD-1’s C2 domain through *CRISPR* |
| PTK447 | *ppk-1(ox874) I;*  *syd-1(wy1320) II; wyIs891 III; oxSi1275 IV* | S2 | *ppk-1(ox874) I*: endogenous ppk-1::GFP::AID  *syd-1(wy1320) II*: endogenous syd-1::FLPon-mScarlet,  *wyIs891 III*: integrated array of unc-87p::FLP (HSNspecific flippase),  *oxSi1275 IV*: Psnt-1::TIR1*(F79G)::F2A::AID::BFP |
| PTK501 | *cla-1(kur27) IV; nrx-1(kur64) V* | 4B, 4D | *cla-1(kur27) IV*: endogenous cla-1::mScarlet  *nrx-1(kur64) V*: endogenous myr::nrx-1(ICD)::skylan-S where the extracellular and transmembrane domains of nrx-1 were replaced with a myristoylation tag using CRISPR, leaving only the intracellular domain (ICD) |

#### **S1 Table. Strain List**
