# Supplementary material for: WormSNAP: A software for fast, accurate, and unbiased detection of fluorescent puncta in C. elegans
Source: PLoS Comput Biol. 2025 Oct 31;21(10):e1013643. doi: 10.1371/journal.pcbi.1013643 (PMC12599913; doi:10.1371/journal.pcbi.1013643)
Supplement: Attachment — Submitted filename: Reviewer_Edits_Rebuttals_final.docx [file pcbi.1013643.s006.docx]

**Reviewer #1:**

 In this manuscript, Tiroumalechetty and colleagues introduce a new software package for the quantitative analysis of fluorescence in the worm nervous system. The authors demonstrate the performance of the software on two test cases: an over-expressed transgene and an endogenously-tagged synaptic protein. They also quantify the ability of their approach to detect differences between wild-type and mutant fluorescence phenotypes as a demonstration of its robustness. Appealingly, the imaging approach is fundamentally 2D rather than the common 1D approach used by multiple groups when analyzing nerve cord synaptic signals. And the software includes multiple approaches to quantification of colocalization of two fluorescence signals from two-color imaging. Overall, this is an impressive effort that could improve the quality of quantitative nervous system imaging in C. elegans if widely adopted. I have a few questions and clarifications for the authors to address but no major issues with this manuscript or with the details of the methodology.

**We thank the reviewer for their supportive statements and will attempt to answer all the questions below.**

1. It is helpful and informative that the authors demonstrated the analysis routines applied to both high signal-to-noise (SNR) data and low SNR data. I was left wondering how well this analysis works as a function of the number of independent worms included in the data set. For instance, do you need significantly more animals in the low SNR case to get the same statistical power as in the high SNR case?

**This is an interesting question. Statistical Power depends on the expected effect size which is independent of the SNR. For example, we can see based on the P-values for the optimal sensitivity in figure 2 that the Observed Statistical Power is lower for the high SNR in terms of differentiating Mild Loss of Puncta Mutant from the Severe Loss of Puncta Mutant but higher in terms of differentiating Mild Loss of Puncta Mutant from the Control. However, the goal of the software is not to carry out statistical analysis but to extract features from confocal images. The expectation is that experimenters carry out their own statistical power analysis before carrying out their experiments.**

I’m not sure how many biological replicates went into the data in Figure 1, but Figure 2 includes what I am guessing are the number of independent images (worms) that went into the ROI count analysis in the various genotypes. Is the 30-40 number fairly typical for the types of analysis performed by this software? Side note - ideally the Fig 2 legend should comment on what the numbers within the bars are referring to.

**Thank you for pointing this out. The number of biological replicates is included in the legend for Figure 1, but the legend for Figure 2 has now been updated to specify that these numbers are biological replicates. The number is fairly typical in the sense that around that many worms are imaged per slide for dataset collection.**

Some commentary on the number of animals needed for reliable estimates of puncta counts, sizes, intensities, etc… could be valuable for future users of this approach.

**Unfortunately, reliability of estimates depends on the variability of the fluorescent markers used, so any commentary would only be valid for these specific markers. Experimenters would be better served by analyzing the standard deviation of the metrics they obtain from the software for their fluorescent marker of choice.**

2. For the colocalization analysis, it was great to see the authors compare different types of colocalization metrics. Figure 4 was a bit confusing, and the legends could be improved by including some more information.

For instance, at the bottom of Fig 4A, what is the significance of showing the ROI perimeters for the >40% threshold case but not showing them for the <40% threshold case where colocalization is scored as true?

**Thank you for this question. The legend has now been clarified specifying that the perimeters shown are only the perimeters of the ROIs assigned as unique. Thus, the ROI not being shown for the <40% threshold indicates that they were not marked as unique.**

And this figure would have been more informative if the reader could see how the overall analysis of colocalization turned out using different colocalization definitions and thresholds rather than just showing the behavior at one or two representative puncta. It’s hard to get a feel for what the final colocalization conclusions are or how the aggregate measures of colocalization would change when switching between methods.

**Good point. The graphs for the Dataset wide Colocalization Metrics have now been added (Fig. C,D).**

3. One minor point on the efforts to look at the impact of noise (and the plots in the Figure 4 supplement). I do not have a feel for how to compare the relative noise levels used in the four noise classes. This would have been more informative if the authors plotted the final output (the normalized correlation metric for example) as a function of noise intensity for each of the four classes of noise rather than at a particular (and perhaps arbitrary or undefined) level of applied background noise. The noise could be increased from 0 to 100% where 100% means the same total intensity is contributed to the cropped image from the noise as from the signal. In this context, the relative robustness of the different metrics to noise class may be more obvious.

**Thank you for this comment. New graphs showing the impact of varied noise levels for thermal (Gaussian) noise, multiplicative (speckle) noise and salt and pepper noise have now been included (Supplementary Figure 4E-G). We were unable to do so for**
**Shot (Poisson) Noise (Supplementary Figure 4D). as it is calculated directly based on the pixel values.**

4. The results section opens with a somewhat abstract discussion of the SNR for the over-expressed and endogenous reporters, and I would have followed better if there was an associated figure with a zoom-in of some representative ROIs (more like what is shown in Figure 4 but emphasizing SNR, Bradley’s method, etc…).

**We are not exactly sure what the reviewer means. Bradley’s method (Local Means Thresholding Algorithm) and comparisons are shown for both High SNR (Figure 1D) and Low SNR (Supp Figure 1A). The threshold has been shown to be robust in low SNR, as shown in Figure 2.**

Minor:
What is the gray dashed line in Figure 1D (local background maybe)? This should be mentioned in the legend or annotated in the figure.

**We have added an annotation indicating that this is the 1-dimensional local means threshold.**

Also, the scale bar is more useful if positioned in the image rather than off to the side where the figure annotations/keys are sitting.

**As part of other suggested edits the raw image was included and the scale bar placed within it.**

**Reviewer #2:**

The manuscript introduces a no-code, user friendly GUI-based MATLAB application tailored for the detection and analysis of fluorescent puncta in C. elegans nerve cord images. The software employs adaptive local means thresholding and watershed segmentation, offering an alternative to traditional global or 1D projection-based methods. Through comprehensive validation—including comparisons with ground truth annotations, metrics, and robustness checks—WormSNAP is shown to outperform existing approaches. It balances accuracy, scalability, and usability, and is designed to be accessible to non-programmers.

Strengths of the work include its methodological rigor, careful benchmarking against other tools, and strong emphasis on user-friendliness, reproducibility, and open access. The GUI design, scalability testing, and inclusion of detailed outputs make WormSNAP well-suited for broad adoption in the C. elegans research community. Limitations include: the tool is constrained to 2D two-channel analysis and relies on a manual cropping pre-processing step, which precludes fully automated pipelines. Additionally, while the validation datasets are well chosen, broader testing across different imaging platforms and organisms would help generalize its utility. Nonetheless, this manuscript presents a meaningful contribution to computational neurobiology and image analysis in model organisms.

This paper appears to be well suited for publication in PLOS Computational Biology, and I support its publication pending revision based on the following comments.

**We thank the reviewer for their supportive statements and will attempt to address all the issues below.**

Here are suggested major changes:
1. The local mean thresholding is generally a great segmentation method, but it often fails when there is another signal in the same FOV after cropping, and many times, and in many cases, cropping alone cannot eliminate other nearby signals.

Also, as presented in Figure 2G, you may find it hard to find a very good sensitivity before testing different sensitivity, which is not ideal for larger dataset analysis (and it is generally faster to manually count puncta if the dataset is small).

I suggest the author consider trying an image filter (perhaps the 2D LoG filter) to highlight those puncta-like structures, and then perform the local mean thresholding. You may find that a precise crop is not necessary after the LoG (or other filter) image enhancement. If this combined method proves to be more effective than local mean thresholding only, please add it to your paper and software. If not, please simply reply with your result to this review. I am also wondering if adding a LoG filter may make the puncta segmentation on typical epifluorescence images possible.

**We thank the reviewer for these comments and made an attempt at Laplacian of Gaussian (LoG) Filter, which was ultimately unsuccessful due to the numerous artifacts in the full images (Fig1A,B Top). Specifically, for the Overexpressed Fluorophore, puncta on the opposite side (due to expression in another neuron’s axon) prevented accurate filtering of the puncta. For the Endogenous Fluorophore, the brightness of the non-specific gut granule fluorescence was too high. Thus we conclude that the generation of crops is an obligate first step. We now note in the manuscript that a series of Fiji macros for speeding up crop generation is available in our repository.**

**The user is indeed expected to try out a few sensitivity settings, as explained in the user guide. However, our software’s speed and ease of visualization allows for quick and easy assessment of different settings, as well as the exclusion of crops with excessive background signal.**

Here are suggested minor changes:
1. In introduction, please add a few paragraphs about why detection and characterization of puncta in C. elegans is an important topic in biology.

**Additional rationale and significance has been added to the introduction.**

2. In Fig. 1Di, I personally cannot tell whether the groundtruth annotation is correct, especially the fourth puncta. Can the author either change the round green circle around each puncta to arrows pointing to them, or manually adjust the gamma of the image to make the figure itself look higher contrast for the audience (but explain this in figure caption), or combine these two methods? The author can also try other methods for readability of the original puncta.

**Thank you for this important and perceptive comment. The Groundtruth annotation for the representative crop and the rest of the dataset was repeated, and the fourth puncta has now been excluded as it was wrongly assigned. The raw crop has now also been shown on top of the rest of the methodologies.**

3. In section of thresholding and segmentation algorithm, please add more explanation to the last formula.

**This has now been done.**

4. Local thresholding often has different performance on areas that are much denser or much sparser than the average signal density. Can author provide some thoughts on this?

**This is a good point. The use of straightened crops that limit analysis to the axon fixes a lot of the issues that occur with variable signal density, as the size of the signal relative to the background is minimized. While we have seen some performance loss in certain strains with very few puncta, using a larger neighborhood size and/or lower sensitivity tends to be enough to fix such issues.**

5. The first paragraph in Results: WormSNAP has been developed to process cropped and straightened images of up to two fluorescent reporters, hereafter simply termed crops. The term of straightened images is not very commonly used in C. elegans community. Please add a quick explanation to it, if the author thinks it is better to add.

**Further description of how the straighten function in Fiji works has now been included.**

6. Section GUI Pipeline, second paragraph, typo Figure 4A.

**This has been fixed.**

7. Figure 3d caption typo, Figure 4 Supp C. Please check the rest of your paper for those kinds of typos, as I see many of these kinds of problems.

**The paper was rechecked for existing typos and those were fixed.**

8. Figure 4: Adding numerical values of Manders’ coefficients and overlap ratios near each example would clarify metric sensitivity. The term “threshold” might confuse readers (intensity threshold vs. overlap percentage). Adding clarification in the caption would help.

**We have attempted to clarify this further.**

9. Please provide some test images of puncta in your GitHub page so people can quickly replicate and verify the segmentation process with WormSnap.

**This is a helpful suggestion. A pre-Analyzed Dataset has now been added to Github.**

**Reviewer #3:**

In this manuscript, the authors provide and describe a MatLab-based software, named WormSNAP, to detect and count fluorescent puncta. The software sets regions of interest (ROI) using adjustable local means thresholding, which the authors show is more robust than previously described methods including global thresholding to define synaptic puncta. The authors showed that software was able to detect the known defects of puncta distributions with severe or mild phenotypes.
The authors also demonstrate the ability to directly visualize the ROIs from two different channels and overlay them. These ROIs can then be used to quantify colocalization of puncta from the separate channels and calculate the Pearson’s Correlation Coefficient, Mander’s Coefficients, and a newly described ROI Overlap Ratio, which determines the fraction of ROIs that are overlapping.
Overall, the software and GIU appear to be user friendly and will aid in determining ROIs that indicate fluorescent puncta seen in confocal images, as well as quantifying these ROIs. I have several comments/suggestions which I hope authors could address before it is published.

**We thank the reviewer for their supportive statements and will attempt to address all the comments below.**

Major Comments:
1. The major limitation of WormSNAP is that, due to how the software exclude noises and background signals such as intestinal autofluorescence, the neuron types that are compatible with the software depend largely on their anatomy; they must be relatively simple, and the puncta must be arranged in a straight line. This may greatly reduce the number of researchers who can benefit from WormSNAP. Can the authors show if WormSNAP can quantify synapses of the HSN neuron in supplemental figure 1? If the use of WormSNAP is limited to the neurons in the nerve cord, the authors should describe it as a major limitation in the discussion section.

**We thank the reviewer for this comment. We have now included expanded analysis of HSN ROIs in Figure S1 to demonstrate that our software is indeed capable of analyzing more complex synaptic arbors such as this one. A bar chart of the normalized ROI counts for the HSN puncta has now been included. Comparison between two sets of images gathered from two different slides of the same genotype is provided, showing that the quantification of the HSN puncta is replicable.**

**A brief description of how crops can be used for non-nerve cord neurons has also been included in the text.**

2. Throughout the manuscript, the authors appear to carefully avoid using C. elegans gene and protein names, especially in the Figures. While the authors implied that this software has the potential to be useful for researchers using other models, this is very much optimized for examining synapses of C. elegans as the name of the software suggests. Not describing the details of the neurons used, the proteins visualized, and the mutant backgrounds used will cause confusion to the main target readers of this manuscript.

**This is helpful feedback. The goal of the figures describing the software is to demonstrate its ability to distinguish between types of fluorophore and mutants with varying effects. While the overexpressed promoter, protein and gene names were included in the methods section of the paper, they weren’t initially included in the figures as they are being used as representative of the general class of fluorophore and phenotype being assessed. However, we have now included them in the main text and figure legends as well.**

3. Related to the above comment, the authors used different genetic backgrounds to represent ‘severe’ and ‘mild’ phenotypes in Figure 2 without defining them in the main text or in the figure, which is very confusing. Also, the phenotypes of nrx-1 and syd-1 mutants that the authors used in Figure 2A-D are qualitatively different and hence not appropriate to be described as mild and severe phenotypes, respectively. Furthermore, the authors compared these mutants only with wildtype. Does the WormSNAP detect the difference between severe and mild mutants?

**Thank you for these comments. The names of the phenotypes for the two reporters have now been changed to “Loss of Puncta Phenotype” for the nrx-1 mutant in the Over-Expression strain and “Clustering Phenotype” for the syd-1 mutant in the Endogenous strain. We also added quantification between these two mutants in the new version of Figure 2, which shows the same general trend in significance as with the WT/Mutant comparisons.**

4. The authors demonstrate, in a step-by-step manner, the robustness of the WormSNAP, in identifying fluorescent synaptic puncta, however, some quantifications lack representative images for viewers to determine the accuracy of the ROI.
a. Figure 1E-G: Only images of overexpressed CLA-1::GFP is shown but the representative endogenous NRX-1:Skylan-S with ground truth annotation, peak finder, global threshold, and local means threshold are not shown.

**This has now been included in the Figure 1 Supplement.**

b. Figure 3D-G: Raw image of C2 and with C2 overlays would be helpful.

**This has now been included as Figure 3, panels F and G.**

c. Figure Supplemental 4A, B: visualization of both fluorescent channels as a visual representation of the Pearson’s correlation,

**This has now been added to Supplement Figure 4A panel, both for the original and cropped images.**

5. Showing the WormSNAP’s ability to detect and segment larger ROIs from a fluorophore with lower signal-to-noise ratio (Ex. Red fluorescent protein tagged with a synaptic vesicle protein) would provide users a greater understanding of the usability of the GUI. This is shown indirectly in Figure 3F and 3G but is not directly addressed.

**This is now explicitly included as Figure 3 panels F and G.**

Minor Comments:
1. Please follow C. elegans genetic nomenclature. For example, the gene names should be italicized, and genes on the same chromones should not have semi-colon in between them.

**This has been fixed.**

2. Figure 1D: Please include the image with no ROI overlay. Some ‘groundtruth’ puncta look almost like ‘noise’ by the authors’ definition.

**This has now been included. The fourth puncta annotated by groundtruth was previously incorrectly assigned and has now been rectified.**

3. Regarding the GUI validation, can the authors suggest a recommended PC specification? Alternatively, the authors could indicate the PC specs they used in this work.

**This has been included in the Github.**

4. Figure 1E-F: Graph titles are confusing, consider using Sensitivity (TPR) and Specificity (TNR) instead of using “/”

**This has been updated.**

5. Supplemental Figure 4C: The names of noises do not match between the Figure and the main text.

**This has now been fixed. We have also now included more description of the type of noise these are meant to model.**

6. There are lots of incorrect figure citations including:
a. GUI Pipeline – “After ROIs are detected in all available channels, users proceed to the Results tab (Supp Figure 3C) to visualize the ROIs and configure output settings. Using the ROI Display panel, users can easily view ROIs. When viewing 2-channel images, users can view ROIs of each channel (Figure 3C)…” Figure 3C does not show ROIs of each channel.
b. Colocalization metrics – “Noise is a major consideration when selecting a correlation metric22. We generated synthetic two-channel datasets based on Channel 1(CLA-1::GFP) of the Overexpressed Synaptic Fluorophore CTRL crops with four different noise types designed to mimic typical confocal microscopy issues: Thermal noise, Shot noise, Salt and Pepper noise, and Speckle noise (Supp Figure 3C).” Should cite Supp Figure 4C.

**Thank you for catching these. We have now fixed incorrect citations as part of general text and figure edits.**

7. Figure panels should be cited in order as first cited. For example, Supp Figure 3F appears before Supp Figure 3D and E.

**We have changed the order of the Supplement Figure 3D-F and changed the text to cite other figures panels in order.**

8. Comparisons of PCC, MC, and ROI Overlap Ratios using actual images of synaptic vesicles and active zone proteins would provide further insight.

**This has been added to Figure 4.**

9. It was unclear what the preprocessing step of “drawing crops” as stated in the discussion entailed, whether it only included straightening of images to include synaptic/axonal regions, or additional work was required.

**A better explanation of this and clarification that drawing crops is done by the Fiji Macro included in the Github Release of the software has now been included in the text.**

10. Comparisons to other simple software or macros (in addition to WormPsyQi) would be helpful in the discussion. Other open-source software that uses free software, such as ImageJ/FIJI has been developed and published to detect and determine synaptic puncta size, fluorescence intensity, and co-localizations.

**We appreciate the comment. The advantage of our software lies in its ability to apply several steps of segmentation, background subtraction, ROI filtering and co-localization. While some macros and simple software do carry out parts of the entire process, they either tend to replicate parts of the same type of analysis (Local Means Thresholding, watershed segmentation,…) as done by WormSNAP or do analysis that has already been discussed as part of Figure 1D (2D Peak analysis, graythreshold). Moreover, most of the macros and simple software are extremely optimized for specific images and require burdensome modification to work with different datasets. Meanwhile, the Crop Drawing macro that generates the input for WormSNAP is compatible with most imaging formats and generates standardized Tiff images.**

**As of writing, only WormPsyQi carries out all the steps of characterization alongside intermediate visualization of ROIs, albeit with an entirely different methodology (ML based axonal recognition), which is why it was selected as a comparison point.**

**While the software is coded in Matlab, it does not require a Matlab license to run, and thus is also free. As one of the main goals of the software is to provide a no-code solution, the software does not require the user the change or edit the code in any way to obtain full functionality. The inclusion of a tailored version of Matlab Runtime with the software also removes the need for users to modify their macros and simple software upon every update of ImageJ/Fiji that impacts the software used. A more thorough explanation of the unique attributes of this software has been added to the text.**
